# MutS*α*’s Multi-Domain Allosteric Response to Three DNA Damage Types Revealed by Machine Learning

**DOI:** 10.3389/fphy.2017.00010

**Published:** 2017-03-30

**Authors:** Ryan L. Melvin, William G. Thompson, Ryan C. Godwin, William H. Gmeiner, Freddie R. Salsbury

**Affiliations:** 1 Salsbury Group, Department of Physics, Wake Forest University, Winston-Salem, NC, USA; 2 Gmeiner Laboratory, Department of Cancer Biology, Wake Forest University School of Medicine, Winston-Salem, NC, USA

**Keywords:** MutS*α*, FdU, cisplatin, carboplatin, decision tree, clustering, molecular dynamics, mismatch repair

## Abstract

MutS*α* is a key component in the mismatch repair (MMR) pathway. This protein is responsible for initiating the signaling pathways for DNA repair or cell death. Herein we investigate this heterodimer’s post-recognition, post-binding response to three types of DNA damage involving cytotoxic, anti-cancer agents—carboplatin, cisplatin, and FdU. Through a combination of supervised and unsupervised machine learning techniques along with more traditional structural and kinetic analysis applied to all-atom molecular dynamics (MD) calculations, we predict that MutS*α* has a distinct response to each of the three damage types. Via a binary classification tree (a supervised machine learning technique), we identify key hydrogen bond motifs unique to each type of damage and suggest residues for experimental mutation studies. Through a combination of a recently developed clustering (unsupervised learning) algorithm, RMSF calculations, PCA, and correlated motions we predict that each type of damage causes MutS*α* to explore a specific region of conformation space. Detailed analysis suggests a short range effect for carboplatin—primarily altering the structures and kinetics of residues within 10 angstroms of the damaged DNA—and distinct longer-range effects for cisplatin and FdU. In our simulations, we also observe that a key phenylalanine residue—known to stack with a mismatched or unmatched bases in MMR—stacks with the base complementary to the damaged base in 88.61% of MD frames containing carboplatinated DNA. Similarly, this Phe71 stacks with the base complementary to damage in 91.73% of frames with cisplatinated DNA. This residue, however, stacks with the damaged base itself in 62.18% of trajectory frames with FdU-substituted DNA and has no stacking interaction at all in 30.72% of these frames. Each drug investigated here induces a unique perturbation in the MutS*α* complex, indicating the possibility of a distinct signaling event and specific repair or death pathway (or set of pathways) for a given type of damage.

## 1. INTRODUCTION

**FIGURE 1 |:**
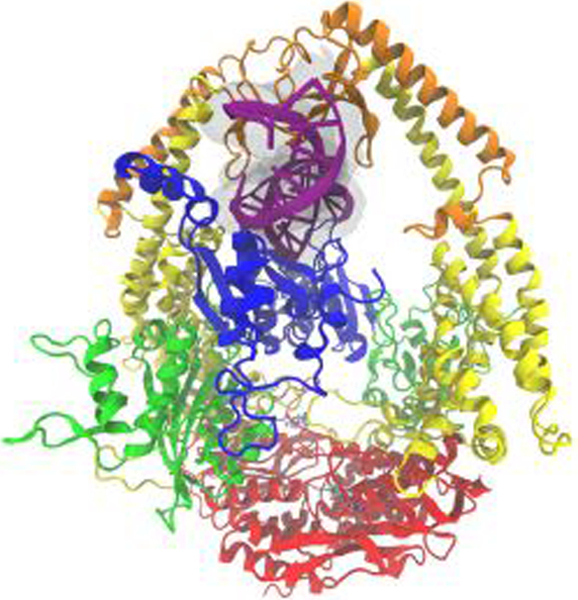
The MutS*α* complex shown here is colored by domain classification. The color coding for the domains Is blue for the mismatch binding domain, green for the connector domain, yellow for the lever domain, orange for the clamp domain, and red for the ATP-ase domain. The nucleic acid strand is colored purple with an additional transparent surface around it for clarity, and the ADP molecules present in the crystal structure [46] are shown in a bonds representation.

In human cells, the most prevalent binding factor is the heterodimer MutS*α*, formed by two MutS homologs (MSH)—MSH2 and MSH6. MutS*α* initiates the repair or apoptotic pathway for mismatched and partner-less nucleic bases [1–7]. The complex contains five functional domains: mismatch binding, connector, lever, clamp and ATPase (Figure 1). Even though binding of damaged DNA occurs in one region, we predict using all-atom microsecond timescale molecular dynamics (MD) simulations and machine learning techniques, that conformational changes and shifts in hydrogen bonding motifs across the entire complex differentiate the heterodimer’s response to carboplatinated, cisplatinated, and flouridated nucleic bases. These results are consistent with several previous studies [8–18] that have shown conformational shifts across interfaces in response to cisplatinated and carboplatinated DNA damage, using short-time (≤10 ns) molecular dynamics with more conventional analysis. However, our present study provides more quantitative details from long-time molecular dynamics (multiple runs of 250 ns) as well as a study of the response to FdU, adding additional insights from novel applications of machine learning techniques. From this investigation, we provide insight into the mechanisms signaling repair or apoptosis by MutS*α*.

Carboplatin [19] [cis-diammine(cyclobutane-1,1-dicarboxylao)-platinum(II)] and cisplatin [20] [cis-diammminechloroplatinum(II)] are both platinum-based anticancer drugs that form platinum-DNA adducts. The anticancer effect of these drugs is that the distortions resulting from such adducts result in cell death [21, 22]. Cisplatin predominately forms G-G crosslinks, whereas carboplatin predominately forms G-X-G crosslinks. In addition to these two types of DNA damage, we also examine MutS*α*’s response to DNA with one base replaced with 5-flouro-2^′^-deoxyuridine (FdU)—a type of DNA-substitution damage—which is likewise cytotoxic [23–37]. For brevity and simplicity we will herein refer to the simulated systems with these three types of DNA damage as “Carbo,” “Cis” and “FdU,” respectively.

The mechanisms of recognition, response and signaling in the mismatch repair (MMR) pathway are not well understood [18, 38, 39]; though, recent computational studies have made progress into the atomic-level details [9, 10, 14, 15]. Here we continue this progress using decision tree learning to identify as few as two key residue interactions differentiating MutS*α*’s response to three types of damage. Additionally, we apply a recently developed unsupervised clustering technique—iMWK-Means with explicit rescaling followed by K-Means [40] (herein *Amorim-Hennig* after the algorithm’s creators)—to identify conformational subtypes adopted by the MSH2-MSH6 complex across simulated responses to the three types of damage. We have previously detailed the particular effectiveness of this clustering techniques on MD simulations of stable systems [41]—such as a structured, functional protein. To bolster confidence in the relatively novel application of these analysis techniques, we compare the specific hydrogen bonding motifs suggested by the decision tree models here to results of previous experimental results and theoretical work. We also point out overlap with and differences from more traditional analysis techniques (see Sections 2 and 4).

## 2. MATERIALS AND METHODS

### 2.1. Force Field Parameters

The CHARMM27 force field [42–44] was used for the entire complex. Due to the relative novelty of the cisplatinated, carboplatinated and fluorinated uracil-containing DNA, additional parameters based on pre-existing carboplatin, cisplatin, and FdU parameters were used in topology generation and simulation [8,17, 23, 30, 45].

### 2.2. Structures

Simulations are based on the X-ray structure of the human MSH2/MSH6 protein complex with heteroduplex DNA, RCSB PDB ID 208B [46]. The structure used in our simulations has a truncated N-terminus. Residues that were excluded by truncating the N-terminus are unstructured, and—while they do play a role in nuclear transport [47]—they do not seem to have any other function. Thus, it is not expected that the truncation of the N-terminus will affect the behavior of the protein complex in these simulations. Hydrogen atoms were added using the hbuild facility of CHARMM [48] since x-ray structures lack hydrogen atoms. In all of the systems, the MSH2 sub-complex contains 855 residues, the MSH6 sub-complex contains 974 residues, the DNA fragments contain 30 bases, and there are two ADP molecules bound to the system.

The cisplatinated, carboplatinated, and matched fluorinated uracil DNA structures were built using the mismatched DNA structure [46] as a template. In the cisplatinated and carboplatinated DNA, the platinum atoms cross-links two adjacent guanine bases. FdU is incorporated into the DNA as a non-canonical base, which impedes replication *in vivo* [23, 29, 31]. These cross-linked structures were fitted into the binding pocket of the MutS*α* complex to maximize the structural overlap with the mismatched DNA structure, followed by rotations and translations to minimize the energy of the initial structure using the coordinate manipulation and energy minimization features included in CHARMM [42–44].

### 2.3. Solvation Conditions

All of these systems were then fully solvated in a cubic water box of size 138Å by 138Å by 138Å with explicit TIP3P water using the VMD package [49, 50]. After solvation, the systems were ionized using the VMD autoionize package to 0.15 mol/L NaCl.

### 2.4. Simulation Configuration

Here, we used ACEMD [50], a simulation program specifically designed for molecular dynamics simulations taking place on GPUs. The molecular dynamics simulations were performed on Acellera Metrocubo workstations with Titan GPUs. These GPUs have 2,688 cores operating at 837 MHz for a theoretical floating point speed of 1.5 Teraflops and allowed for 11.5 ns per day on a single GPU for simulations of MutS*α*.

To begin our simulations, the water molecules in all systems were minimized for 100 cycles of conjugate gradient minimization, with a small harmonic force constant on all protein atoms. This minimization ensures that the TIP3P water atoms were distributed in a physical way, which prevented the introduction of perturbations due to any initial un-physical configurations of water atoms such as steric clashes. All systems then underwent a small 250 ps simulation in order to reach thermal equilibrium using Berendsen pressure regulations with isotropic position scaling [43, 51]. These simulations used Berendsen pressure with ACEMD default parameters (target pressure of 1.01325 Bar relaxation time of 400 fs) and Langevin damping (0.1/ps) for temperature control [50].

Equilibration was performed by assigning atoms velocities from a Boltzman distribution for a given temperature every 1,000 cycles in 25 K increments from an initial temperature of 0 K to a final temperature of 300 K. Production runs were performed on all systems using 4 fs timesteps, which required hydrogen mass repartitioning, as implemented in ACEMD [50]. To calculate VdW and electrostatic forces, we used a cutoff distance of 9Å with a switching distance of 7.5Å. For longer range interactions, we calculated electrostatics using a smooth particle mesh Ewald (SPME) summation algorithm [52, 53]. We ran two simulations of 250 ns each system (i.e., 500 ns per system), saving data every 2,500 time steps (10 ps).

### 2.5. Processing and Analysis

To expedite memory and time-intensive analysis techniques, we resampled all trajectories, keeping every tenth frame (i.e., 100 ps per frame). We also removed water and counter-ions prior to analysis. To focus on internal motions of the protein-DNA complex, we aligned all frames in a given trajectory to the trajectory’s first frame via rigid body rotations and translations to minimize the RMSD of protein alpha carbon positions. This alignment was carried out with the RMSD trajectory tool in VMD [49].

#### 2.5.1. Hydrogen Bond Detection

We concatenated trajectories of common atoms from all six resulting simulations into one trajectory file (15,000 frames comprising 1.5 *μ*s). In this concatenated trajectory, we detected hydrogen bonds between polar atoms using the Python [54] package MDAnalysis [55, 56]. As input parameters, we defined a hydrogen bond as having a maximum heavy atom to heavy atom distance of 3.2Å and a maximum heavy atom to hydrogen to heavy atom angle of 120 degrees—an intermediate strength hydrogen bond [57]. We focused on various subsets of the complex’s hydrogen bonds, as detailed in the Section 3.

We parsed the output of the MDAnalysis hydrogen bond detection algorithm into a Pandas DataFrame [58] for easy conversion to a comma separated value file compatible with Matlab and the Statistics and Machine Learning Toolbox therein. For those wishing to reproduce our hydrogen bond detection and parsing, we have made our processing scripts and the underlying data available for free online via figshare [59].

#### 2.5.2. Decision Tree Learning

Using the output of the hydrogen bond detection and processing scripts [59] and Matlab’s Statistics and Machine Learning Toolbox, we trained a binary classification tree using the presence of residue-residue hydrogen bonds in each frame as input features and the name of damage type associated with each frame as the responses. We have used the same terminology as the Matlab documentation to describe this process so that readers who investigate the Matlab decision tree manual pages and help files will be able to easily match our usage to Matlab documentation.

The input *features* were in the form of a two-dimensional matrix with a row representing a trajectory frame and a column representing a possible hydrogen bond interaction. Each entry in the matrix was a 1 or 0 indicating the presence or absence of the specific hydrogen bond in that particular trajectory frame. The input *responses*, or known correct labels, were the name of the damage in the simulation from which a given frame came. Specifically, each frame from simulations with cisplatinated DNA was labeled “cis;” each frame from simulations with carboplatinated DNA was labeled “carbo;” and each frame from simulations with FdU-substituted DNA was labeled “FdU.”

The Matlab command “fitctree” outputs a binary classification decision tree with branching nodes that show the binary—yes or no—presence of features that lead to a specific response. In the case of our data sets, the decision tree differentiates the three types of damage based on the presence or absence of certain hydrogen bonds (see Section 3). This machine learning technique allowed us to determine which residue-residue interactions distinguished MutS*α*’s response to the three types of simulated damage.

In addition to supplying the most likely label at the terminus (*leaf*) of each path in the decision tree, Matlab estimates the probability of each type of damage for any frame that follows this path. In this case, we used a uniform prior probability in calculating estimated probability for each leaf (the default option in Matlab’s “fitctree” function). Therefore, estimated probabilities reported here were calculated by [the number of frames in a given system that follow a given path] divided by [the total number of frames across all systems that follow that path].

For training these classification trees, we used all default parameters, specifying only the features (hydrogen bond trajectories) and responses (damage types) as inputs. Matlab 2016a—used here—outputs an interactive decision tree plot that allows users to explore various levels of *pruning. Pruning* reduces the depth of the decision tree, collapsing data points in the removed level into the place in the next highest level that creates the least amount of *loss* (classification error). Using the text-based commands “prune” and “loss” in the Matlab Statistics and Machine Learning toolbox, a user can calculate the percent of mislabeled frames given a certain pruning level. We use these commands to simplify decision trees given various error tolerances (see Section 3).

**FIGURE 2 |:**
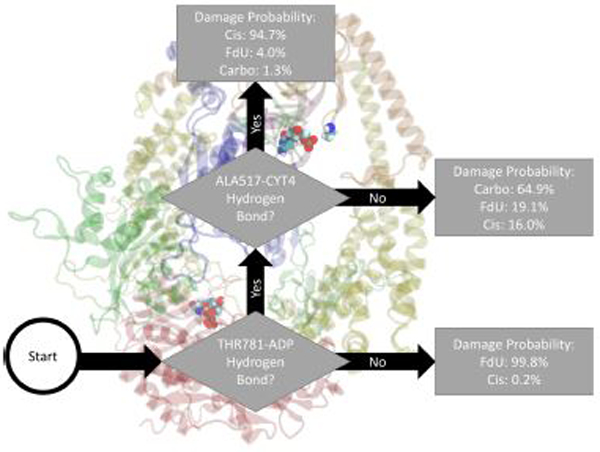
Based on the presence of two hydrogen bonds Thr781 on MSH6 to an ADP molecule and Ala517 on MSH2 to Cyt4 on the damage DNA our decision tree, fitted on hydrogen bonds between the protein and the bound nucleic acids (including ADP), correctly labels the type of damage in 80% of MD frames (and mislabels the other 20%). How to read all such figures: The flow chart shown here indicates the damage type predicted by two-level decision tree as the damage type with the highest estimated probability (see Section 2) along with the probability of other types of damage. That is, decision output from Matlab and pruned to 2 remaining levels would label a frame with both a Thr781-ADP and a Ala517-Cyt hydrogen bond as “Cis.” In the MD data, 94.7% of frames with these two hydrogen bonds were in the Cis trajectories, 4.0% were in FdU trajectories, and 1.3% were in Carbo trajectories. Residues (or bases) involved in the interactions described in the flow chart are in VDW representation. The description of the interaction is shown directly adjacent to residues involved so that the reader may easily see the domain and specific location of the relevant residues.

As an example of how to read the flow chart figures reporting the output of the decision trees, Figure 2 indicates the damage type predicted by two-level decision tree as the damage type with the highest estimated probability along with the probability of other types of damage. That is, decision output from Matlab pruned to 2 remaining levels would label a frame with both a Thr781-ADP and a Ala517-Cyt hydrogen bond as “Cis.” Using these highest likelihood labels, Matlab would correctly label 80% of the frames (20% *loss* or *classification error*). In the MD data, 94.7% of frames with these two hydrogen bonds were in the Cis trajectories, 4.0% were in FdU trajectories, and 1.3% were in Carbo trajectories. Residues (or bases) involved in the interactions described in the flow chart are in VDW representation. The description of the interaction is shown directly adjacent to residues involved so that the reader may easily see the domain and specific location of the relevant residues.

Note that these trees were fitted on all data points (as opposed to training on one subset and validating on another), as our goal in using this machine learning technique was to uncover in a programmatic, reproducible manner which residue interactions distinguished the types of damage in our simulation data. We then investigate the error created by various levels of pruning (see Section 3) to find two or three key residues that are key in differentiating the types of damage. For these particular analysis goals, there would be no benefit to separate training and validation sets.

For those wishing to reproduce our analysis, we have scripted this analysis process and made those scripts along with the underlying data available only for free online (https://figshare.com/articles/Scripts_and_Data_for_MSH26_Damage_Response_article/4003266) via figshare [59]. For those interested in the theoretical details of decision tree learning, there are many recent, excellent reviews and introductory chapters [60–63].

#### 2.5.3. Non-parametric Clustering

Using a recently developed unsupervised learning technique, which is made effectively non-parametric by the the use of sensible defaults, we explored overlap and dissimilarities of MutS*α*’s conformational response to the three types of damage simulated here. Amorim-Hennig [40] clustering requires the user to select a distance metric in the form of a Minkowski Weight [40, 64]. Here we chose a Minkowski weight of 2, corresponding to Euclidean distance. We have previously detailed the particular effectiveness of this clustering method on MD data [41] and have made Python scripts for applying this method to MD trajectories available for free online via figshare [65].

#### 2.5.4. Correlated Motion and PCA

Using Pearson Correlation, we calculated a correlated motion matrix of protein alpha carbons for each system and for the concatenated trajectory of all simulations. By diagonalizing the resulting matrices, we performed Principal Component Analysis (PCA), which reduces the number of coordinates from 3 × number of atoms to just a few components capturing the majority of dynamic variance in the simulations (see Section 3). By projecting the original trajectories onto the eigenvectors representing the two highest-variance (largest eigenvalues) principal components, binning the the projections with a two-dimensional histograms, and converting to free energy values with

$$\Delta G=-kT\ln\frac{p}{p_{0}}$$

(where *k* is Boltzmann’s constant, *T* is temperature (here 300 K), *P* is the population of a given bin and *P*_0_ is the population of the highest populated bin), we estimate a free energy landscape for the protein’s dynamics.

To aid comparison across systems, we first calculated principal components for the concatenated trajectory of two simulations for each of three damage types (6 simulations) to create a common basis set. We then projected coordinates from each system individually, producing a free energy landscape for each of the three damage types in a common space.

#### 2.5.5. RMSF

For additional validation of the the machine-learning-based classification (based on hydrogen bonds) and clustering analysis (based on atomic coordinates) and to examine the changes in relative flexibility of each MutS*α* and DNA residues in response to varying the type of DNA damage, we calculate the per residue Root Mean Square Fluctuation (RMSF) of each backbone alpha carbon using

$$\text{RMSF}=\sqrt{\frac{1}{N}\sum_{t_{j}=1}^{N}{\left({\vec{r}}_{i}\left(t_{j}\right)-{\vec{r}}_{i}^{\prime }\right)}^{2}}$$

where *N* is the total number of frames, *t*_*j*_ is an instance in time, ${\vec{r}}_{i}$ is the position of atom *i*, and ${\vec{r}}_{i}^{\prime }$ is the position of that atom in the average structure.

#### 2.5.6. Coordinate Parsing and Distance Calculations

For RMSF analysis and non-parametric clustering we read atom positions into computer memory and performed all necessary distance calculations using the MDTraj Python package [66]. For PCA calculations, we read atomic coordinates into Matlab 2016a using MatDCD (www.ks.uiuc.edu/Development/MDTools/matdcd/) and produced free energy plots using in-house Matlab scripts, which we have made avaialble online via figshare [67].

### 2.6. Structure Visualization

We produced all structure images using VMD and Tachyon [68]. For figures indicating conformational uncertainty using shadows, we individually rendered the representative (solid) structure and each frame in the shadows. We then combined the output image files using Pillow, a fork of the Python Image Library. The images provide both a representative conformation for cluster as solid with shadows showing the full width of the distribution so as to avoid deceiving a viewer into thinking the cluster is a single conformer. The representative structure in each visualization is that with the smallest RMSD from the average of all structures in the cluster. Additional technical details of this visualization style and the underlying statistical reasoning for producing them has been previously detailed [69]. Our scripts for producing such images are available online via figshare [70].

## 3. RESULTS

### 3.1. Hydrogen Bonds and Decision Trees

Fitting a decision tree to the binary hydrogen bond trajectory of interactions between protein residues and nucleic bases (including adenine on the two ADP residues) from the concatenated data of all systems yielded a decision tree with 37 levels of depth that correctly labels the type of damage in 99.82% (i.e., 0.18% loss) of MD frames (Supplementary Figure 1). Pruning by 12 levels yielded a tree with 1% loss (Supplementary Figure 2), and pruning by 31 levels yielded 5% loss (Supplementary Figure 3). That is, using binary knowledge of the presence of at most 9 hydrogen bonds we correctly label the damage type in 95% of MD frames. In fact, with knowledge of just two sets of interacting residues, our decision tree—pruned to two levels of depth—correctly labels the damage type in 80% of frames (Figure 2). These two interactions that have the largest influence in differentiating the type of DNA damage are (1) hydrogen bonding between Thr781 on MSH6 and an ADP molecule and (2) Ala517 on MSH2 and Cyt4 on the damaged DNA.

For comparison, a similarly pruned decision tree fitted on only hydrogen bonds between the protein and damaged nucleic acid double strand correctly labeled 74% of frames (Supplementary Figure 4). A similarly pruned decision tree fitted on only hydrogen bonds between the protein and the two ADP molecules correctly labeled 69% of frames (Supplementary Figure 5).

**FIGURE 3 |:**
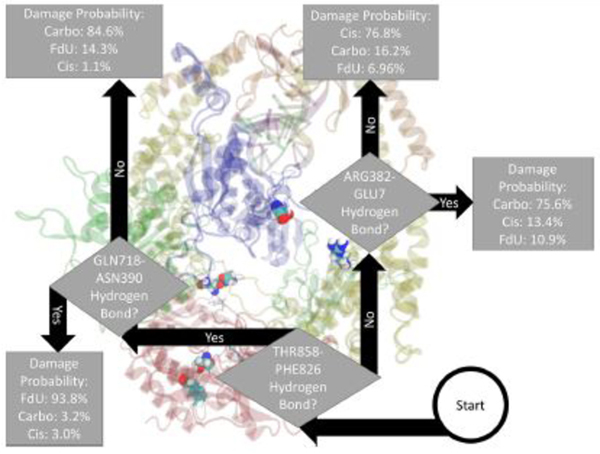
Based on the presence of three hydrogen bonds Thr858 on MSH6 to Phe826 on MSH2, Asn390 on MSH6 to Gln718 on MSH2, and Glu7 on MSH6 to Arg382 on MSH2 our decision tree, fitted on hydrogen bonds between the two protein monomers, correctly labels the type of damage in 82% of MD frames (and mislabels the other 18%). For guidance on how to read this figure, see Section 2 and the caption of Figure 2.

Fitting a decision tree on the binary hydrogen bond trajectory of interactions between the two protein monomers (MSH2-MSH6) from the concatenated data of all systems yielded a decision tree with 39 levels of depth that correctly labels the type of damage in 99.91% (i.e., 0.09% loss) of MD frames (Supplementary Figure 6). Pruning by 17 levels yielded a tree with 1% loss (Supplementary Figure 7), and pruning by 30 levels yielded 5% loss (Supplementary Figure 8). That is, using binary knowledge of the presence of at most 4 hydrogen bonds we correctly label the damage type in 95% of MD frames. In fact, with knowledge of just three sets of interacting residues, our decision tree—pruned to two levels of depth—correctly labels the damage type in 82% of frames (Figure 3). The three interactions that have the largest influence in differentiating the type of DNA damage are (1) hydrogen bonding between Thr858 on MSH6 and Phe826 on MSH2, (2) Asn390 on MSH6 and Gln718 on MSH2, and (3) Glu7 on MSH6 and Arg382 on MSH2.

**FIGURE 4 |:**
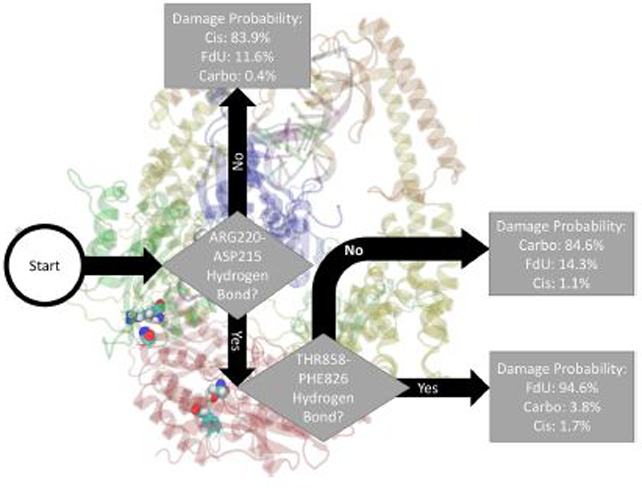
Based on the presence of two hydrogen bonds Thr858 on MSH6 to Phe826 on MSH2 and Arg220 on MSH6 to ASP215 also on MSH6 our decision tree, fitted on hydrogen bonds between any protein residues and any other residue or base (including another protein residue), correctly labels the type of damage in 86% of MD frames (and mislabels the other 14%). For guidance on how to read this figure, see Section 2 and the caption of Figure 2.

Fitting a decision tree on the binary hydrogen bond trajectory of interactions between protein residues and any other residue (including another protein residue) or base (damaged double strand or either ADP molecule) from the concatenated data of all systems yielded a decision tree with 21 levels of depth that correctly labels the type of damage in 99.96% (i.e., 0.04% loss) of MD frames (Supplementary Figure 9). Pruning by 10 levels yielded a tree with 1% loss (Supplementary Figure 10), and pruning by 15 levels yielded 5% loss (Supplementary Figure 11). That is, using binary knowledge of the presence of at most 3 hydrogen bonds we correctly label the damage type in 95% of MD frames. In fact, with knowledge of just two sets of interacting residues, our decision tree—pruned to two levels of depth—correctly labels the damage type in 86% of frames (Figure 4). The three interactions that have the largest influence in differentiating the type of DNA damage are (1) hydrogen bonding between Thr858 on MSH6 and Phe826 on MSH2, (2) Asn390 on MSH6 and Gln718 on MSH2, and (3) Glu7 on MSH6 and Arg382 on MSH2.

### 3.2. RMSF

**FIGURE 5 |:**
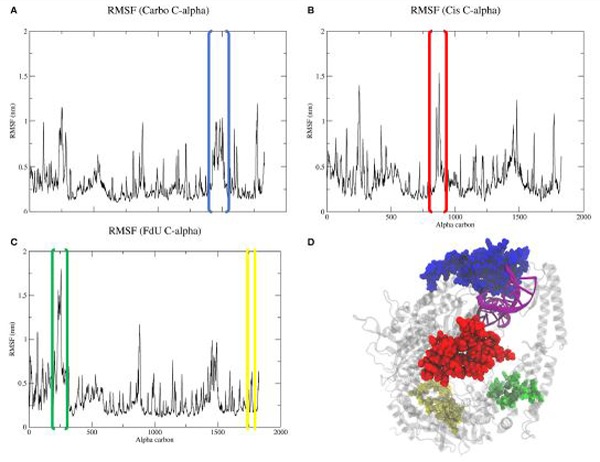
Comparison of RMSF **(A–C)** of protein alpha carbons reveals generally similar regions of flexibility in each system. However, the Cis **(B)** and FdU systems **(C)** have distinct peaks, indicating uniquely mobile regions. While the highlighted region in the Carbo system **(A)** is roughly the same magnitude in value as the other systems **(B,C)**, there are more residues within the peaked region in the Carbo system **(A)**. This same region also has a slightly narrower but taller peak in the Cis system **(B)**. Additionally, we see stabilization in the ATP-ase domain of MSH6 in response to FdU-substituted DNA **(C,D)**. For reference, residues in the highlighted portions of the plots are shown with matching colors on the crystal structure **(D)** with the nucleic acid colored purple for emphasis. These regions are residues between **(A)** Ala579 and Lys670 in the lever and clamp domains of MSH6 for the Carbo system, **(B)** residues between Lys9 and Met49 in mismatch binding domain of MSH6, and **(C)** residues between Lys235 and LEu270 in the connector domain of MSH2 and between Gln919 and Ala959 of the ATPase domain of MSH6 for the FdU system.

Calculating the root mean square fluctuation of protein alpha carbons in each simulated system indicates that damage type correlates to to a change in mobility of certain MutS*α* regions (Figure 5). While all three systems show a spike around residue 1450 (Asn599 in the the clamp domain of MSH6), when MutS*α* is exposed to carboplatinated DNA, this mobility expands to MSH6 residues between Ala579 and Lys670, which involves both the clamp the lever domains (Figures 5A,D). Cisplatinated DNA evokes a similarly unique response, causing a spike in mobility of MSH6 residues between Lys9 and Met49 in the mismatch binding domain (Figures 5B,D). In the Cis system, we also see a spike for MSH2 residues between Lys235 and Leu270 (Figures 5B,D) in the connector domain. This spike is larger for the FdU system (Figures 5C,D). As an additional response to FdU, we also see stabilization of residues in the ATP-ase domain of MSH6.

### 3.3. PCA and Binding Site Structures

**FIGURE 6 |:**
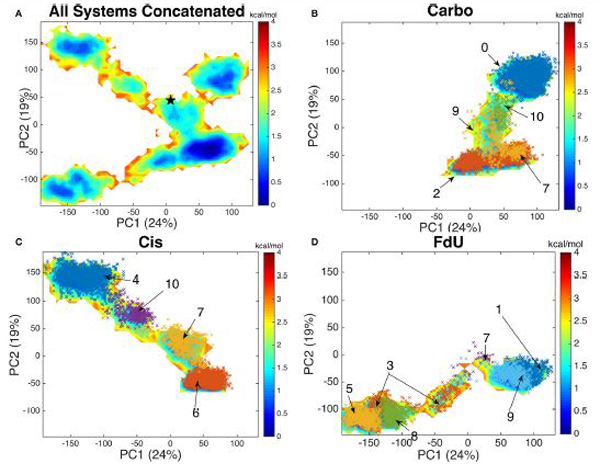
By projecting each system onto the common basis **(A)** set formed by decomposing the covariance matrix of the concatenated trajectory of all systems, we see that each system—**(B)** Carbo, **(C)** Cis, and **(D)** FdU—enters a distinct region of the free energy map while having a common well. The crystal structure (initial coordinates for each simulation) is marked with black star in the first panel near coordinates (10, 50). Each color-coded and labeled set of x’s is one structural cluster, based on residues near the damaged DNA. The cluster numbers correspond to those in Figure 7. We plot these additional points here to demonstrate how similarly binding site clustering and a PCA histogram partition of the trajectories are. From this comparison, we see xsthat binding site motions are the primary cause of the various free energy wells in PCA1–2 space.

Using an aligned trajectory of coordinates concatenated from all simulations (6), we created a common basis set for projecting coordinates of all systems into a two-dimensional space. We use the two principal components that explain the highest amount of variance (totaling 43%). Projecting both the concatenated trajectory of all simulations and separately the coordinates from each simulated system (Carbo, Cis and FdU), we see that each system explores a distinct segment of the reduced-dimension space (Figure 6).

The free energy surface created by projecting the concatenated trajectory of all simulations (Figure 6A) is of course, fictitious, as no single system could visit all coordinates on the surface. That is, no system would be ergodic in the space used to create that surface. However, by creating this landscape using coordinates from all trajectories, we can see the kinetic overlap—or lack thereof, among the three systems. For reference, we mark the coordinates of the crystal structure on the fictitious free energy landscape—coordinates (10, 50) on Figure 6A.

By comparing the free energy landscape of the concatenated trajectory (Figure 6A) to the free energy landscape of the individual systems (Figures 6B,C), we see that while there is a shared segment of the landscape near the coordinates of the crystal structure, each system does explore a unique portion of the space.

By plotting the coordinates of Amorim-Hennig heavy atom clusters of residues with any atom within 10 angstroms of the DNA onto the (alpha-carbon-coordinate-based) PCA1–2 space of each system (Figures 6B,C), we see that the binding site clustering forms a partition similar to that of the distinct wells seen in the free energy landscape. From this overlap of partitions we infer that the the motion of protein residues near the damaged DNA is responsible for the various free energy wells the system enters.

**FIGURE 7 |:**
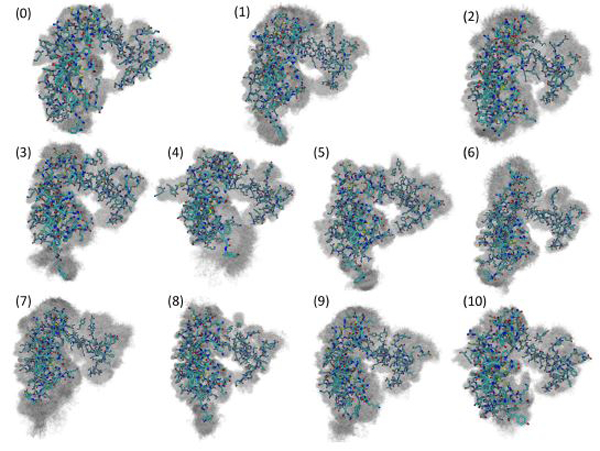
Visualization of structural clusters of residues with any atoms within 10 angstroms of the bound nucleic acid reveals MutSα’s local response to various types of DNA damage. The cluster numbers here correspond to the labels in Figure 6. Amorim-Hennig clustering placed frames from the Carbo simulations in clusters 0 (45.86% of Carbo frames), 2 (39.90%), 7 (10.50%), 9 (0.02%), and 10 (3.72%). Frames from Cis simulations were placed in clusters 4 (40.36% of Cis frames), 6 (36.22%), 7 (16.18%), and 10 (7.24%). Frames from the FdU simulations were placed in clusters 1 (36.40% of FdU frames), 3 (16.08%), 5 (22.00%), 7 (0.70%), 8 (10.94%), and 9 (13.88%). By plotting frames from these clusters on the estimated free energy landscape using the dominant principal components (Figure 6), we see that binding site motions are the primary cause of the various free energy wells in PCA1–2 space. The solid structure in each cluster visualization is the trajectory frame with the smallest RMSD from the average of all structures in the cluster. Shadows are all frames in the cluster, so that the reader may gauge the width of the distribution [69].

Visualizing the representative conformations of these binding site clusters and along with the underlying structural distributions (Figure 7), we see that each system’s binding site enters distinct conformations for large portions of their respective trajectories. We also see that portions of these trajectories have significant structural overlap across systems—in terms of the residues near the damaged DNA. Amorim-Hennig clustering placed frames from the Carbo simulations in clusters 0 (45.86% of Carbo frames), 2 (39.90%), 7 (10.50%), 9 (0.02%), and 10 (3.72%). Frames from Cis simulations were placed in clusters 4 (40.36% of Cis frames), 6 (36.22%), 7 (16.18%), and 10 (7.24%). Frames from the FdU simulations were placed in clusters 1 (36.40% of FdU frames), 3 (16.08%), 5 (22.00%), 7 (0.70%), 8 (10.94%), and 9 (13.88%).

### 3.4. Correlated Motions

**FIGURE 8 |:**
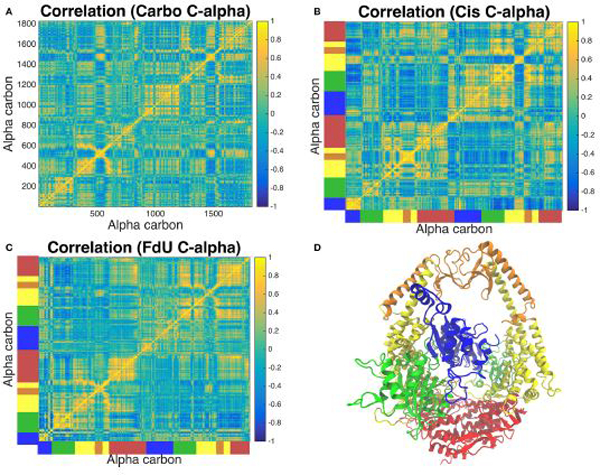
Analysis of correlated motions of common alpha carbons across all three systems reveals the response of the protein’s long-range residue communication network to the three types of DNA damage. The Carbo system **(A)** exhibits pockets of strong correlation in the lever, clamp and ATP-ase domains. Correlation of these regions involves more residues and is of greater intensity in the Cis system **(B)**. In this system, we also see correlations across monomers emerge in the connector and mismatch binding domains, which is greatly reduced in the FdU system **(C)**. However, we see greater correlation in the connector domain in MSH2 (but not MSH6) in the FdU system **(C)**. For reference, the protein crystal structure colored by domain is shown in **(D)** with the same colors as in Figure 1. These colors are also used on the axes of **(B,C)** to indicate the domain that corresponds to the residue number in **(A)**.

Correlated motion analysis reveals a distinct communication pattern associated with each type of DNA damage (Figure 8). In response to carboplatinated DNA, we see small regions of relatively strong correlation among residues of the lever, clamp and ATP-ase domains (Figure 8A). These correlated regions broaden to more residues and increase in relative strength in these domains in response to cisplatinated DNA along with the emergence of strong correlations across monomers in the connector and mismatch binding domains (Figure8B). While these correlations across the two monomers are greatly reduced in the FdU system, we see stronger correlations in the connector domain of MSH2 in response to FdU substitution (Figure 8C).

### 3.5. Phe Stacking

**FIGURE 9 |:**
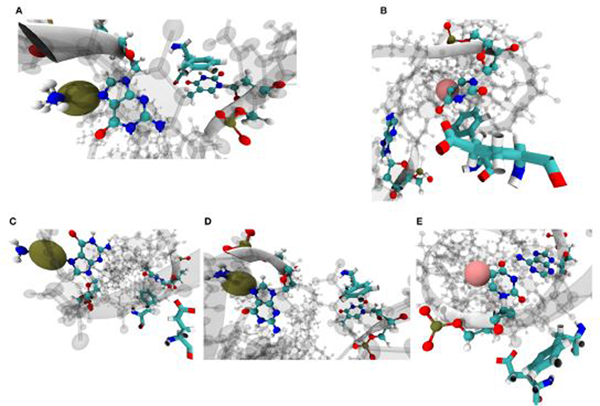
Amorim-Hennig clustering on heavy atoms of Phe71 and Glu73 on MSH6, previously implicated in mismatch recognition and repair [8, 9, 11, 71, 72], reveals the local structural response of the protein to the three types of DNA damage. In cluster 0 through 3 **(A–D)**, we see the aromatic ring on phenylalanine stacking with either the damaged base **(B)** or the the complimentary base to the damaged base **(A,C,D)**. Phe and Glu enter the conformation shown in **(A)** in 12.28% of Carbo frames, 46.90% of Cis frames and 1.72% of FdU frames; **(B)** in 11.12% of Carbo frames, 8.52% of Cis frames, and 62.18% of FdU frames; **(C)** in 36.88% of Carbo frames, 43.98% of Cis frames, and 5.38% of FdU frames; **(D)** in 39.58% of Carbo frames and 0.58% of Cis frames; **(E)** in 0.14% of Carbo frames, 0.02% of Cis frames, and 30.72% of FdU frames. In these visualizations, the damaged DNA structure is taken from the cluster’s representative frame. Damaged bases and their complimentary bases are shown as solid CPK representation with all other nucleic bases shown with shadow. Platinum atoms in frames from Carbo and Cis systems are colored a dark gold and in VDW representation. In FdU systems, the fluorine atom is shown in VDW representation for emphasis and is colored pink. Phe71 and Glu73 are shown in solid bonds representation. Camera perspective is adjusted in each panel to show Phe stacking **(A–D)** or lack thereof **(E)**.

Phe71 and Glu73 on MSH6 have been previously implicated in mismatch recognition and repair [8, 9, 71, 72]. Amorim-Hennig clustering on heavy atoms of these two residues revealed that the phenylalanine residue assumes a conformation suited for stacking with base complimentary to the damaged base predominantly in simulations of Carbo and Cis systems. Such a Phe conformation for stacking occurs in in 88.74% of Carbo frames, 91.46% of Cis frames, and 7.1% of FdU frames. A conformation suited for stacking with the damaged base occurs in roughly 62.18% of FdU frames (Figure 9).

## 4. DISCUSSION

Across all the analysis techniques detailed above, we consistently see that each of the three drugs—carboplatin, cisplatin and FdU—induces a distinct perturbation in MutS*α*. Each drug altered the dimer’s hydrogen bond network in ways easily distinguished by knowledge of a few key residue interactions. All three drugs altered residue mobility across multiple domains in distinct ways. MutS*α* explored a different part of its free energy landscape based on the type of damage in the bound DNA. Patterns of long and short range correlated residue motion changed dramatically across the various types of damage. Three types of nucleic acid interaction with Phe71 on MSH6 emerged, two of which occur in Carbo and Cis systems with the third occurring almost exclusively in the FdU system. In this section, we will first discuss the implications of these results for each drug followed by general insights about the MSH26 complex gained in our investigation.

### 4.1. Response to Carboplatinated DNA

In this study, we saw multiple kinetic and structural factors distinguishing MutS*α*’s response to carboplatinated DNA. The presence of hydrogen bonds between Thr781 on MSH6 and ADP, Thr858 on MSH6 and Phe826 on MSH2, and Glu7 on MSH6 and Arg382 on MSH2 were key in distinguishing the Carbo system from the other types of simulated damage (Figures 2–4). In part, these results indicate a given damage type induces a specific interaction between the protein ATP-ase domain and nearby ADP molecules. The importance of ATP/ADP binding as a response to DNA mismatch and damage has been experimentally demonstrated [4, 6, 11, 72–77], and the force field parameters used in our simulations have been previously validated as consistent with these experimental results [8–10, 17, 18, 78]. In this present study, we add—byway of binary classification trees—the insight of which particular residues in the ATP-ase domain (and other domains) are most influential in distinguishing the type of damage.

The decision tree methodology discussed in this work cannot by itself indicate a causal link between these hydrogen bonds and response to damage. However, the fact that Thr781 on MSH6, Thr858 on MSH6 and Phe826 on MSH2 so clearly separated out the Carbo systems suggest that these residues would be good initial candidates for future mutation studies, examining the change in repair and apoptosis signaling when these residues are not present. From the decision tree fitted on our MD data, we would expect mutations of these residues to confer resistance to carboplatin.

Calculation of alpha carbon RMSFs (Figure 5A) indicates that MutS*α*’s response to carboplatinated damage involves mobilizing more protein residues near the DNA than does its response to the other two types of damage. Correlated motion analysis is consistent with the RMSF results, as we see more residues involved in the highly correlated region of the MSH6 mismatch binding domain along with a circular blip of highlighted correlated residues near coordinates (1,500, 600) and (600,1,500) in Figure 8, which are residues the clamp and lever domains of both monomers. This cluster of highly correlated residues is larger and has greater intensity than in the correlation matrices of the other two systems. From these results, we see that carboplatinated DNA has a greater local effect on protein residues than the other two systems in which we see more long range, allosteric effects.

Experimental work has shown that MutS attempts to bend DNA through motion of the mismatch binding and clamp domains as part of the mismatch recognition process [1, 79]. Mobilization of these same residues in the simulated Carbo system shows overlap between the response to carboplatinated DNA and mismatched DNA. This overlap indicates that MutS*α* may sometimes enter a repair-signaling conformation in response to carboplatin rather than a death-signaling conformation. The PCA free energy landscapes in Figures 6A,B lend credence to this hypothesis. The Carbo system has a shallow free energy well near the coordinates of the crystal structure, which is known to be in a mismatch-repair-signaling conformation [46]. That is, we see the Carbo system entering conformations similar to the known mismatch response. This observation is consistent with previous computational studies that showed a higher overlap of carboplatin-mismatch recognition conformations (55%) than of cisplatin-mismatch overlap (45%) [9,10].

Additionally, PCA and Amorim-Hennig clustering (Figures 6B, 7) indicate that while there is some structural and kinetic overlap with the Cis and FdU systems, carboplatinated-DNA-bound MuS*α* enters a region of conformation space and principal-component space distinct from those regions entered in the other two systems. That is, carboplatinated DNA induces unique structures and kinetics. By examining the overlap of the PCA free energy wells with clustering on binding site residues, we see that the motion of binding site residues is primarily responsible for the kinetic variance seen in PCA free energy landscape. Available experimental data suggests that there are distinct differences in the protein complex’s response to carboplatin compared to cisplatin [19, 22, 38, 80, 81]. However, experimental atomic-level details of kinetic differences are not yet available for comparison to the MD predictions reported here.

More specifically, from clustering on heavy atoms of Phe71 and Glu73 on MSH6 we see both the Carbo and Cis systems distinguished from the FdU system by the stacking of Phe71’s aromatic ring with the nucleic base complimentary to the damaged base. Phe71 is in a stacking conformation in 88.74% of trajectory frames, indicating that carboplatinated DNA is highly likely to induce this conformation. In previous experimental and computational studies of MutS*α*’s response to mismatched DNA, Phe71 was observed to stack with one of the mismatched bases [8, 9, 11, 71, 72]. In the presence of carboplatinated DNA, we see Phe71 stacking primarily with a base complimentary to the damage base.

### 4.2. Response to Cisplatinated DNA

The binary classification trees describing the hydrogen bonding patterns in our simulations indicate that MutS*α*’s response to cisplatinated DNA is distinguished by the presence of hydrogen bonds between Thr781 on MSH6 and ADP, and Ala517 on MSH2 and Cyt4 on the damaged DNA. The decision tree fitted on inter-subunit interactions (Figure 3) between the two protein monomers indicates that the Cis system is distinguished more by a lack of protein-protein interactions than by their presence. That is, exposure to cisplatinated DNA disrupts the hydrogen bonds between the two protein monomers. This result is consistent with experimental work showing that cisplatin either altered or removed most inter-subunit interactions [8]. This previous study indicates that the loss or change of inter-subunit interaction in the ATP-ase domain was particularly pronounced with cisplatinated DNA, consistent with the decision pathway to Cis in Figure 3. Furthermore, mutation studies focusing on the ATPase domain have demonstrated it plays a key role in the damage response and repair pathways [11].

While the Cis system was differentiated more by the absence of certain hydrogen bonds than the presence, the protein residues Thr781 and Ala517 in these hydrogen bond pairs would be our suggested candidates for an initial mutation study. Additionally, in a previous computational study with simulations on a shorter—nanosecond—timescale, the Thr781 residue was implicated in MutS*α*’s response to mismatched and cisplatinated DNA [14], indicating that this residue has a role to play across multiple timescales.

RMSF analysis indicates that exposure to cisplatinated DNA increases the mobility of residues in the mismatch binding domain. A previous mutation study supplemented by MD calculations suggested that structural response to cisplatinated DNA is localized to the ATPase domain [8]. However, our calculations predict the most dramatic change in the mismatch binding domain, and in fact, Cis has the most instances and highest valued cross-domain correlation of alpha carbon motion (Figure 8B). This disagreement of results is likely due to the computational power available at the time of the previous study, which produced 1.6 ns of MD data. A decade later, we are able to produce 300-fold more data, observing much longer timescale events. On a 1ns timescale, the allosteric effects of cisplatin may be localized to the ATP-ase domain; however, we predict on the 100 ns timescale that these effects spread to multiple domains.

Another experimental study shows structural changes across the connector and lever domains in response to cisplatinated DNA [11]. Our prediction of increased correlated motion in these and other domains in the Cis system is consistent with the experimental results. Other experimental studies have indicated the MSH2 is not involved in apoptotic signaling in response to cisplatinated DNA [11, 82]. In fact, one mutation study that removed the MSH2 ATP-ase domain entirely still observed MSH-induced cell death in response to cisplatinated DNA [82], lending confidence to our prediction of MutS*α* response to cisplatin across other subunits in addition to the ATP-ase domain.

We also see that, similar to the Carbo system, the Cis system explores a portion of the PCA-based free energy landscape near the crystal structure (Figures 6A-C). These results are, again, consistent with previous calculations indicating 45% overlap between cisplatin-induced structures and mismatch-induced structures [9, 10]. From these free energy landscapes we also see some overlap of the Carbo and Cis systems near the coordinates of the crystal structure. Therefore, we infer that similar to the Carbo system, MutS*α* sometimes enters a repair-signaling conformation in response to cisplatinated DNA. However, the PCA free energy landscapes and heavy-atom clustering on protein residues near the damaged DNA indicate that both systems enter unique conformations (Figures 6, 7). The dominant free energy wells corresponding to binding site clusters 2 and 0 (Figure 6B) in the Carbo system are structurally and kinetically distinct (compare Figure 6 and Figure 7) from the dominant free energy wells of the Cis system—clusters 6 and 4, consistent with experimental studies indicating distinct responses to carboplatin and cisplatin [19, 22, 38, 80, 81].

The key phenylalanine residue enters a stacking conformation with the base complementary to the cisplatin-containing base in 91.46% of trajectory frames with cisplatinated DNA (Figure 9). Stacking of Phe71 with the strand opposite the damaged base in response to cisplatinated DNA was predicted by a previous computational work [8]. Experimental mutation of this Phe residue to Ala indicated that the Phe residue is not necessary for cisplatin-induced apoptosis [8]. Therefore, whatever the *in vivo* role of Phe71 stacking with the complimentary base in response to cisplatined DNA might be, it is clearly not critical for death signaling as suggested by early simulation work [8].

### 4.3. Response to FdU-Substituted DNA

The binary classification trees describing the hydrogen bonding patterns in our simulations indicate that MutS*α*’s response to FdU-substituted DNA is distinguished by the presence of hydrogen bonds between Thr858 on MSH6 and Phe826 on MSH2, Gln718 and Asn390, and Arg220 and and ASP215 (Figures 2–4). We suggest these residues as initial candidates for a future mutation study. Additionally, across all three decision trees, FdU consistently had the highest likelihood, indicating that FdU was the most cleanly separated and easily distinguished system in terms of hydrogen bond motifs. This ease of differentiation makes intuitive sense, as FdU-substitution is a distinctly different type of damage from the metal-DNA cross-linked adducts of cisplatin and carboplatin.

Exposure to FdU-substituted DNA increases the mobility of residues in the connector domain of MSH2 and decreases that of the ATP-ase domain of MSH6 (Figure 5C). The increased mobility of the connector domain is consistent with the localized increase in correlated motions (Figure 8C). We also see that the MSH2 connector domain has greater overall correlation with all domains relative to the other two systems (Figure 8), further indicating the MSH2 connector residues are key in MutS*α*’s response to FdU. This MSH2-focused, increased-mobilization response to FdU but not carboplatin or cisplatin is consistent with experimental work showing that MSH2 is not involved in the response to platinum DNA damage [82]. Furthermore, the original work reporting the crystal structure for MutS*α* bound to mismatched DNA indicates conformational shifts in the connector domain [46]—similar to the increased flexibility of the connector domain predicted by our MD calculations—in response to FdU (Figures 5C,D). That is, the FdU-response of MutS*α* appears to be repair-signaling. This inference is consistent with other experimental studies showing MMR is able to repair fluorouracil-containing DNA [27, 34, 83–87].

At first blush, this inductive conclusion seems at odds with the known cytotoxicity of flouridated uracils [23–32, 34–37]. However, there are two facets to consider. First, the experimental studies showing that MMR pathways can repair FdU-substituted DNA suggest cytotoxicity is caused by secondary events—such as depletion of the thymine pool or futile cycling in thymineless conditions—leading to apoptosis [23, 26–31, 34, 83, 87, 88]. Additionally, our heavy-atom clustering of Phe71 and Glu73 indicates that Phe enters a stacking conformation similar to that observed in MMR. We observe this mismatch-repair-like stacking with the damaged base—as opposed to its complement [8, 9, 71, 72]—in roughly 2/3 of FdU frames with no stacking in the remaining 1/3. From these calculations, we infer that FdU does not always cause repair signaling, but may be inducing a death-signaling conformation roughly 1/3 of the time.

If FdU is inducing a repair-signaling conformation, our PCA free energy landscapes and binding site clustering results indicate it is distinct from the mismatch-repair-signaling conformation in the crystal structure [46] used here for initial coordinates. The free energy landscapes show no exploration of the region near the PC1–2 coordinates of the crystal structure (Figures 6A,C). Therefore, we would predict that if solved, a crystal structure of MutS*α* bound to FdU-substituted DNA would have significant structural differences from that of the structure bound to mismatched DNA despite the fact that both systems are capable of signaling a repair pathway.

### 4.4. Conclusions

Across all three systems, we saw evidence of MutS*α* entering repair conformations for some fraction of MD frames. These results do not necessarily mean that the heterodimer would signal repair in response to all types of damage; instead, they may indicate that the protein is searching for the appropriate conformation to respond to the type of damage, and that search involves exploring repair conformations in addition to death-signaling. From all analysis techniques, we see indications that each system explores its own portion of conformation space (Figures 2–7) and engages in unique kinetics (Figures 6, 8), indicating distinct perturbations induced by each type of damage.

Our MD calculations and binary decision tree fittings establish hydrogen bond motifs as clear distinguishing characteristics of MutS*α*’s response to each type of DNA damage. With knowledge of just 2 or 3 hydrogen bonds, these decision trees can correctly label the damage type up to 86% of MD frames, further indicating that each type of damage is inducing its own, unique response and likely signaling a unique cellular response pathway (or set of pathways) *in vivo.*

We also see from Amorim-Hennig clustering that Phe stacking with bases on the bound damaged DNA has some role to play in responding to each type of damage. Though, that role is not ultimately critical to the signaling of the repair or death pathways. We also find the similarity between the phenylalanine’s response to FdU and its known response to mismatched DNA striking (Figure 9), especially given that PCA indicates that FdU never explores the mismatch-repair-signaling portion of PC1–2 space (Figure 6).

The predictions from MD calculations presented here are consistent with the available experimental data. However, through the “computational microscope” [89, 90] of MD, we both contribute atomic level details of known MutS*α* damage responses and suggest areas of further investigation for future experimental studies on aspects of damage response that have not been previously investigated. We also present novel applications of decision tree learning and the recently developed Amorim-Hennig clustering technique to MD data, hoping to inspire not only further research on MutS*α*’s damage response but also wider usage of these machine learning techniques for systematically and reproducibly analyzing macromolecular data.

## Supplementary Material

supplemental
