# Supplementary material for: MutSα’s Multi-Domain Allosteric Response to Three DNA Damage Types Revealed by Machine Learning
Source: Front Phys. Author manuscript; Available in PMC 2020 Jan 14. (PMC6959842; doi:10.3389/fphy.2017.00010)
Supplement: supplemental [file NIHMS1063499-supplement-supplemental.pdf]

Supplementary Material: Allosteric Reaction Across Multiple Domains Differentiate MutSα's Response to Three Types of DNA Damage as Revealed by Machine Learning

Ryan L. Melvin [1], William G. Thompson [1,+] William H. Gmeiner [2], and Freddie R. Salsbury, Jr [1,*]

*Correspondence:
Freddie R. Salsbury Jr
salsbufr@wfu.edu

1 FIGURES

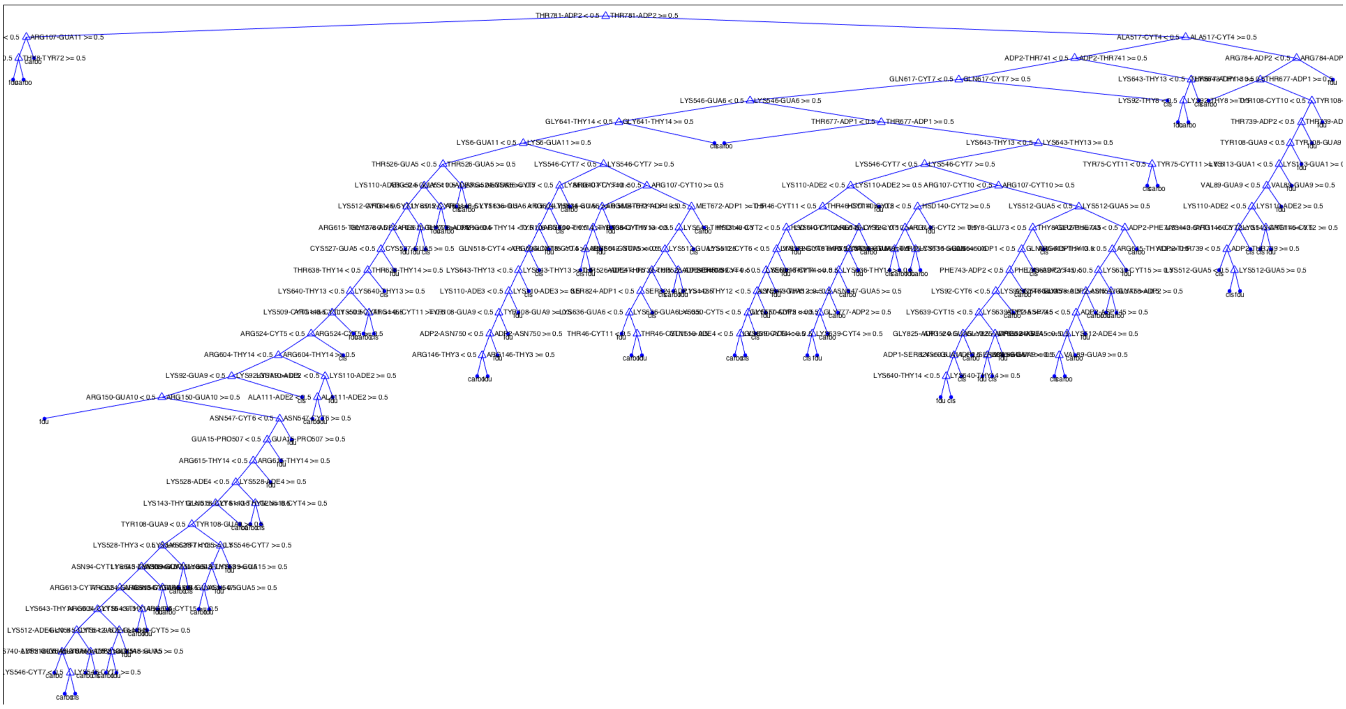

Supplementary Figure 1. Fitting a decision tree on the binary hydrogen bond trajectory of interactions between protein residues and nucleic bases (including adenine on the two present ADP residues) from the concatenated data of all systems yielded a decision tree with 37 levels of depth that correctly labels the type of damage in 99.82% (i.e, 0.18% loss) of MD frames.

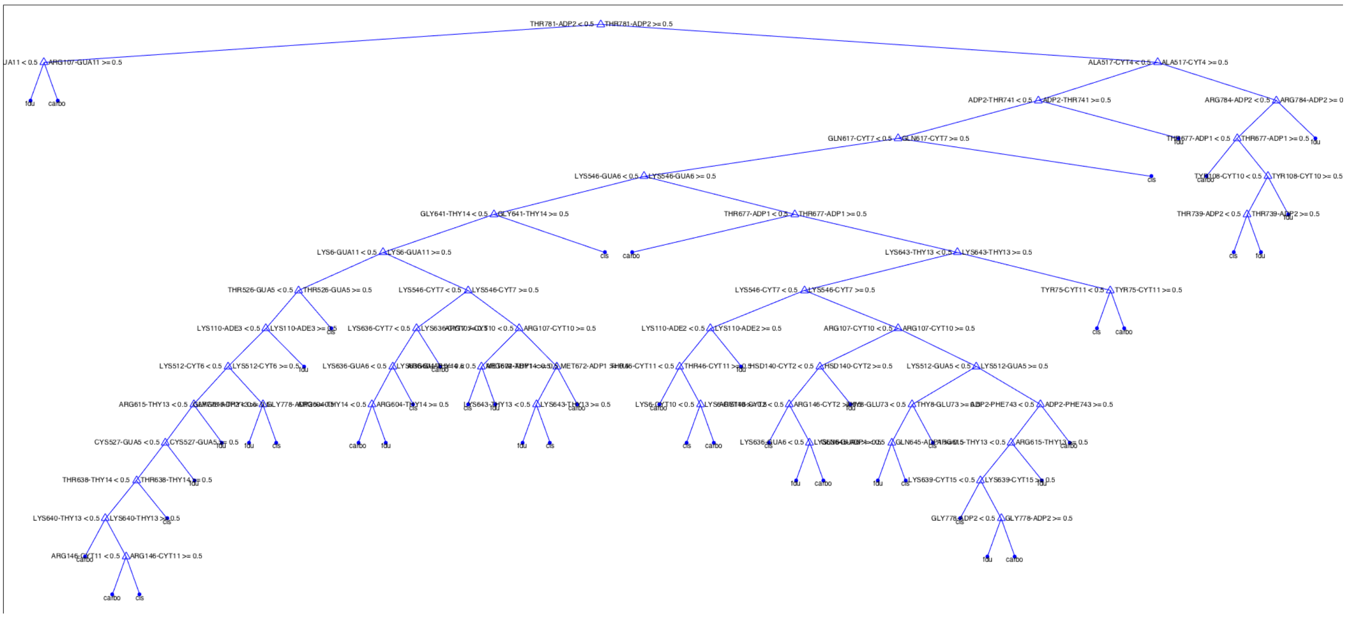

Supplementary Figure 2. Pruning the decision tree in Supplementary Figure 1 by 12 levels yielded a tree with 1% loss.

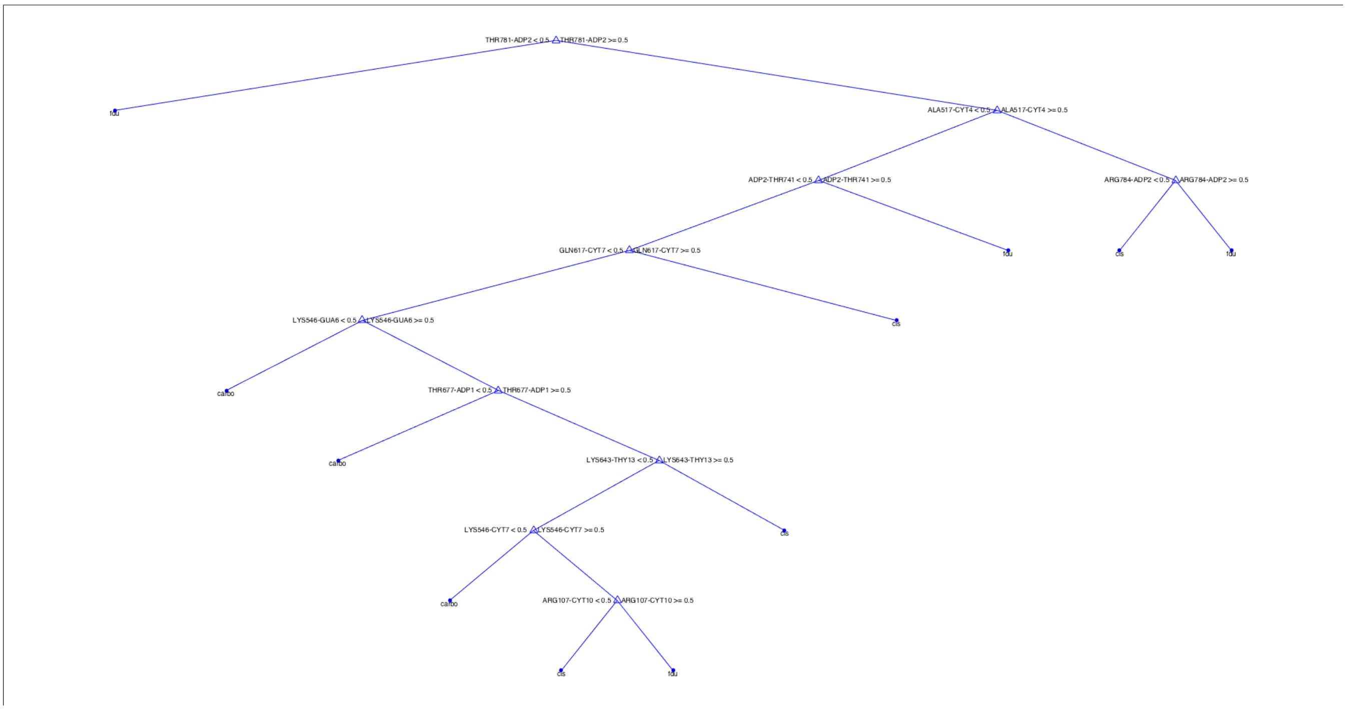

Supplementary Figure 3. Pruning the decision tree in Supplementary Figure 1 by 31 levels yielded a tree with 5% loss.

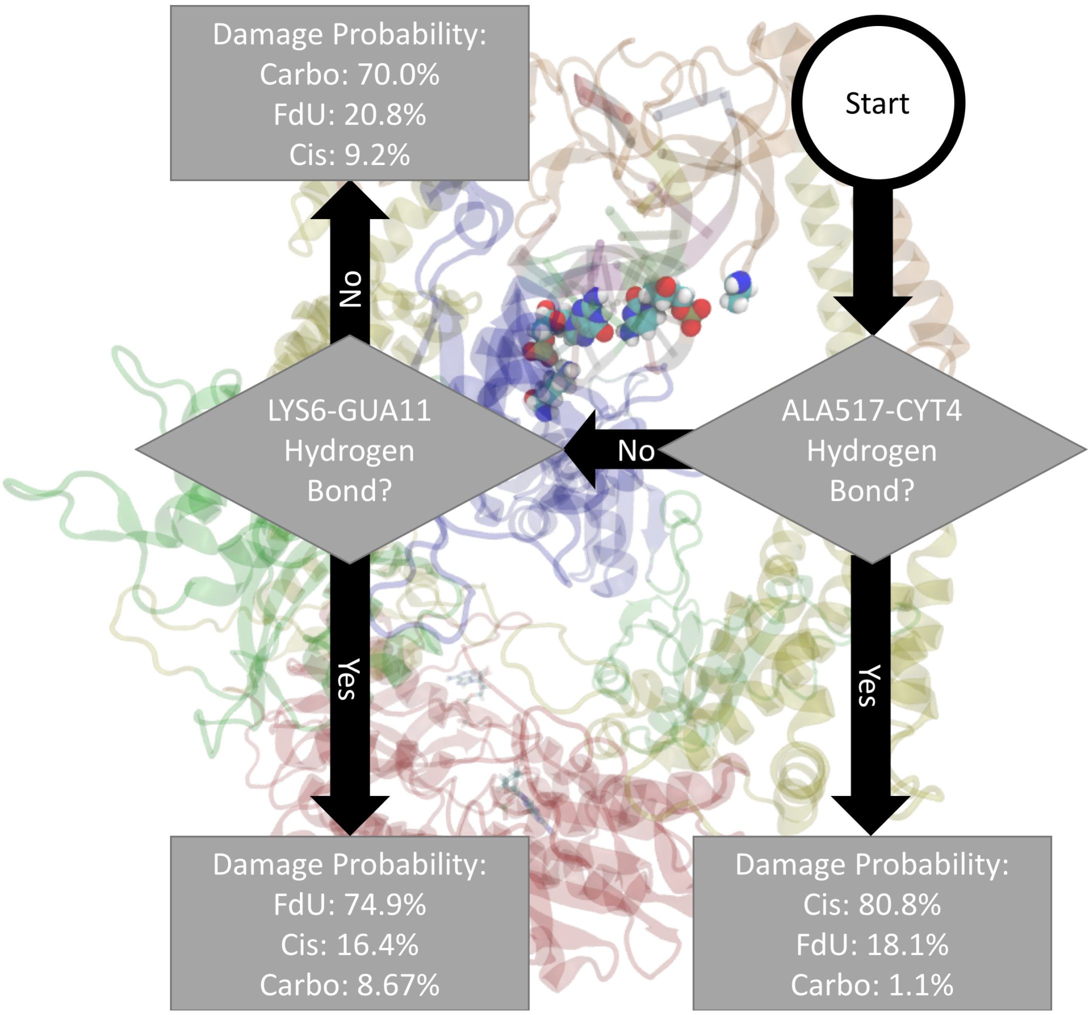

Supplementary Figure 4. Based on the presence of two hydrogen bonds Ala517 on MSH2 to Cyt4 on the damaged DNA and Lys6 on MSH2 to Gua11 on the damaged DNA our decision tree, fitted on hydrogen bonds between the protein and the damaged DNA, correctly labels the type of damage in 74% of MD frames (and mislabels the other 26%). For guidance on how to read this figure, see Methods and the caption of Figure 2.

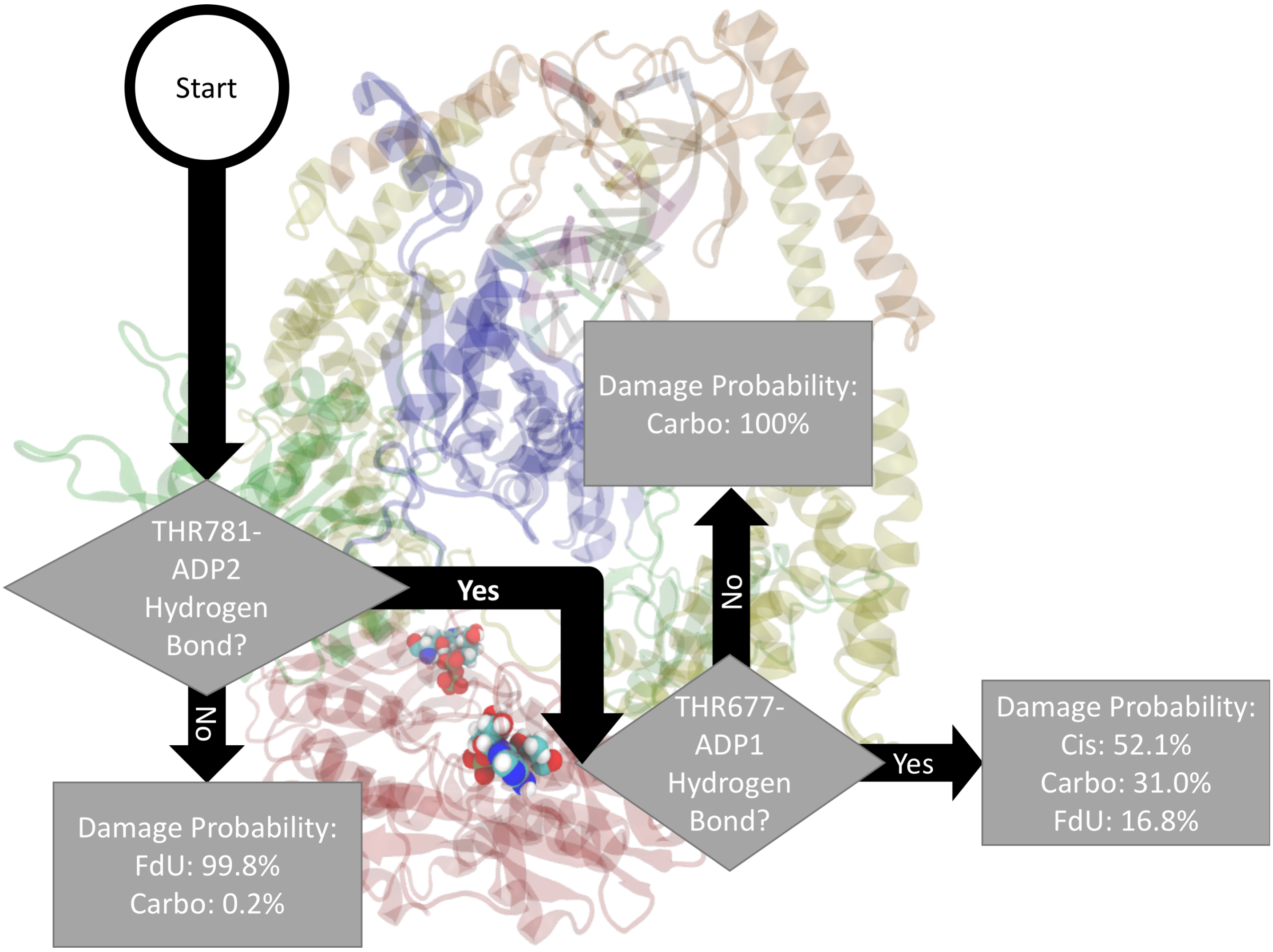

Supplementary Figure 5. Based on the presence of two hydrogen bonds Thr781 on MSH6 to one ADP molecule and Thr677 on MSH2 to the other ADP molecule our decision tree, fitted on hydrogen bonds between the protein and ADP molecules, correctly labels the type of damage in 69% of MD frames (and mislabels the other 31%). For guidance on how to read this figure, see Methods and the caption of Figure 2.

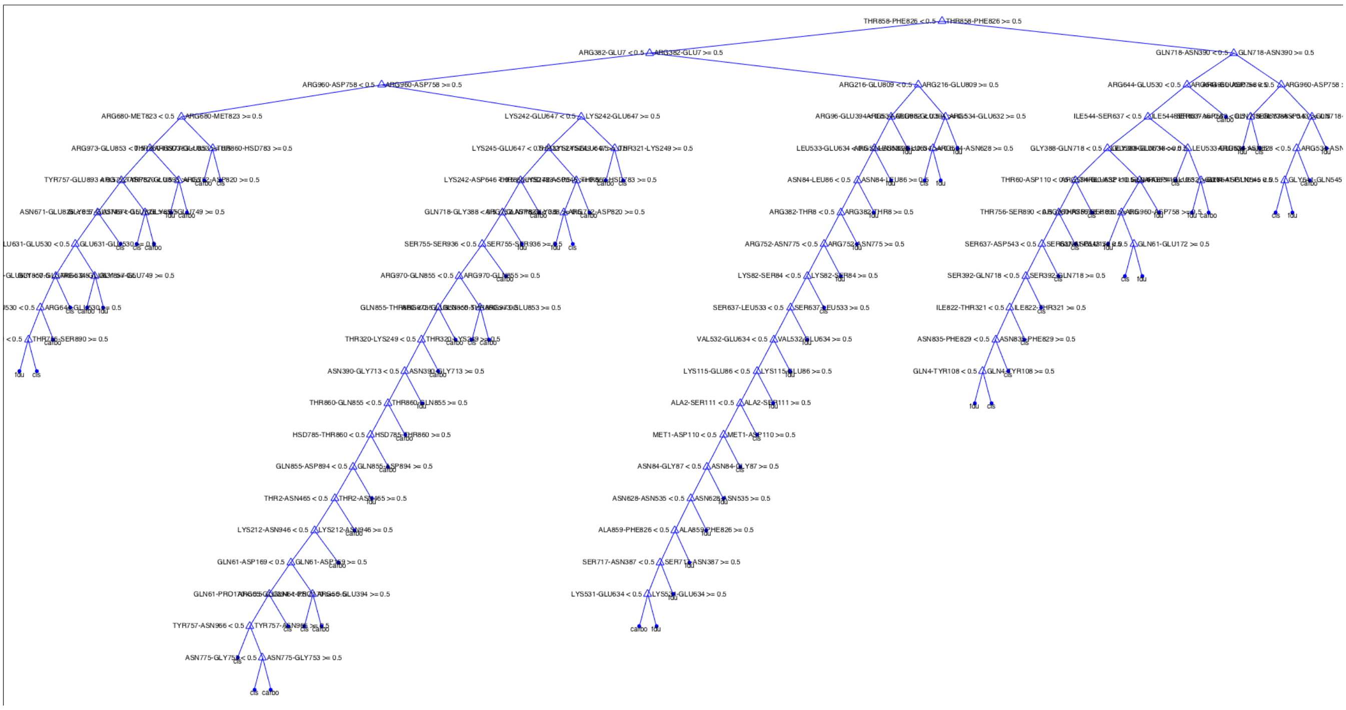

Supplementary Figure 6. Fitting a decision tree on the binary hydrogen bond trajectory of interactions between the two protein monomers (MSH2-MSH6) from the concatenated data of all systems yielded a decision tree with 39 levels of depth that correctly labels the type of damage in 99.91% (i.e, 0.09% loss) of MD frames.

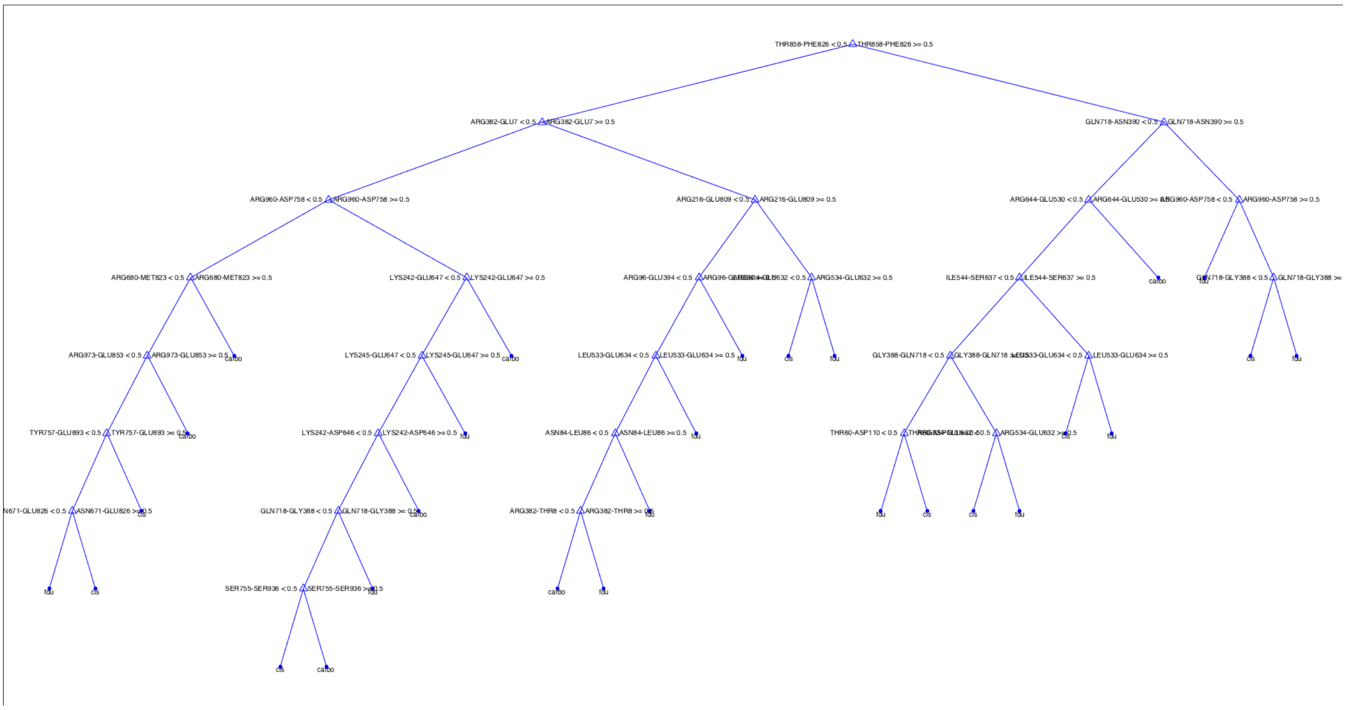

Supplementary Figure 7. Pruning the decision tree in Supplementary Figure 6 by 17 levels yielded a tree with 1% loss.

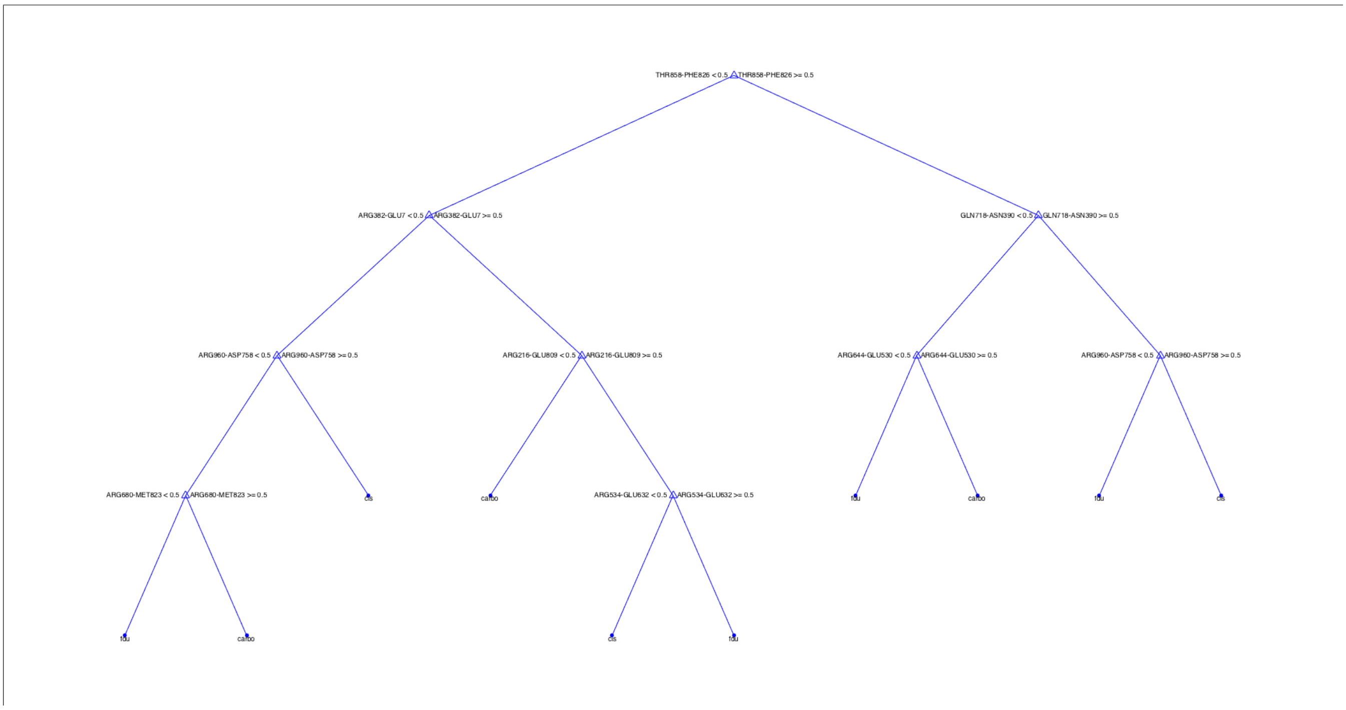

Supplementary Figure 8. Pruning the decision tree in Supplementary Figure 6 by 30 levels yielded a tree with 5% loss.

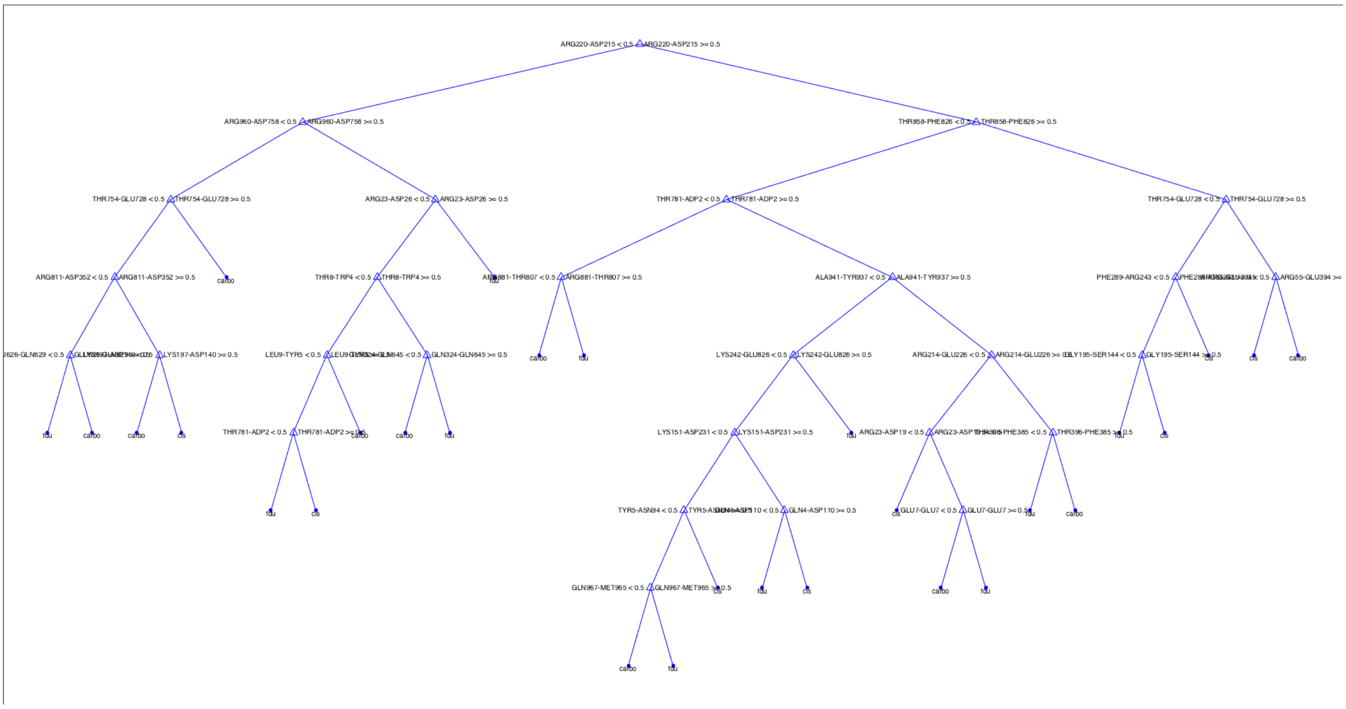

Supplementary Figure 9. Fitting a decision tree on the binary hydrogen bond trajectory of interactions between protein residues and any other residue (including another protein residue) or base (damaged double strand or either ADP molecule) from the concatenated data of all systems yielded a decision tree with 21 levels of depth that correctly labels the type of damage in 99.96% (i.e, 0.04% loss) of MD frames.

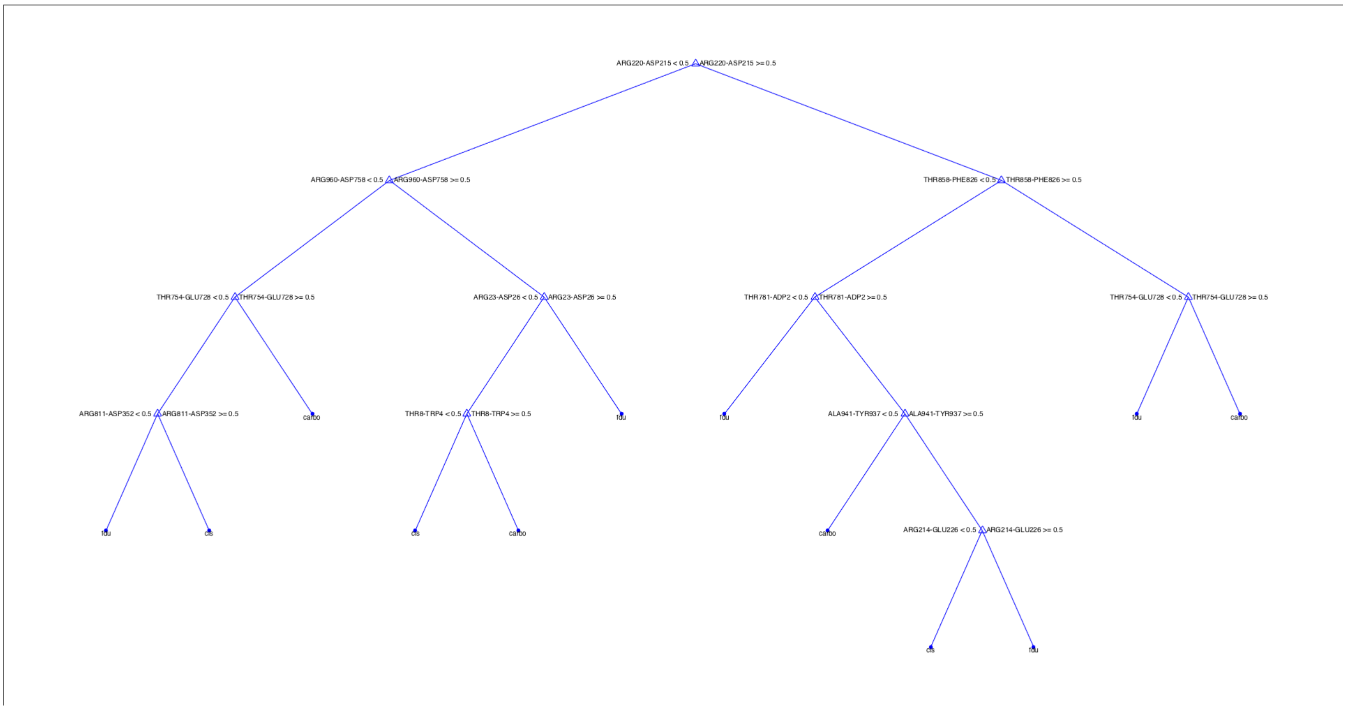

Supplementary Figure 10. Pruning the decision tree in Supplementary Figure 9 by 10 levels yielded a tree with 1% loss.

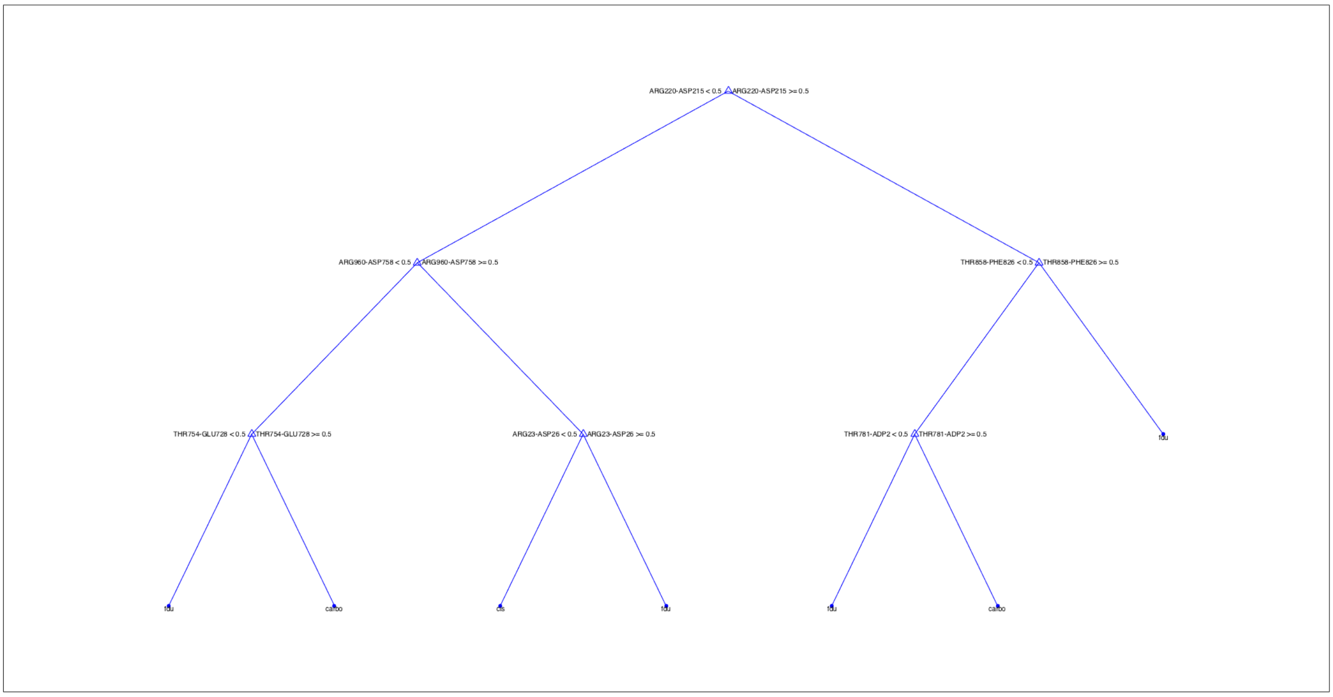

Supplementary Figure 11. Pruning the decision tree in Supplementary Figure 9 by 15 levels yielded a tree with 5% loss.
